# Development of a Pharyngeal Residue Level Assessment Index Using Artificial Intelligence (AI) Acoustic Analysis: A Study Protocol

**DOI:** 10.7759/cureus.78358

**Published:** 2025-02-01

**Authors:** Yoshitaka Shimizu, Tamayo Takahashi, Aya Oda, Serica Imamura, Eiji Imado, Utaka Sasaki, Hisanobu Kamio, Maho Suyama, Ryo Uetsuki, Shinichiro Ohshimo, Nobuaki Shime, Hiroshi Hanamoto

**Affiliations:** 1 Department of Dental Anesthesiology, Hiroshima University Graduate School of Biomedical and Health Sciences, Hiroshima, JPN; 2 Department of Dental Anesthesiology, Division of Oral and Maxillofacial Surgery and Oral Medicine, Hiroshima University Hospital, Hiroshima, JPN; 3 Department of Oral and Maxillofacial Surgery, Hiroshima University Graduate School of Biomedical and Health Sciences, Hiroshima, JPN; 4 Department of Emergency and Critical Care Medicine, Hiroshima University Graduate School of Biomedical and Health Sciences, Hiroshima, JPN

**Keywords:** artificial intelligence, aspiration, electronic stethoscope, pharyngeal residue, sound monitoring

## Abstract

The swallowing function is often compromised immediately after general anesthesia owing to the effects of anesthetic agents. Consequently, pharyngeal residue may accumulate, which increases the risk of aspiration during the perioperative period. Therefore, we designed a single-arm, open-label study, developing an artificial intelligence (AI)-based acoustic analyzer for quantifying pharyngeal residues and evaluating its efficacy. A sample of 30 patients aged ≥18 years scheduled for jaw deformity surgery will be enrolled in this study. Immediately after tracheal tube extubation, adventitious sounds from pharyngeal residues, such as saliva and blood, will be measured and quantified using an AI acoustic analysis system. Subsequently, the residual pharyngeal fluid will be suctioned and quantified by measuring the change in container weight before and after collection. The primary outcome measure will be the comparison of adventitious sounds before and after pharyngeal suction, and the secondary outcome will be the correlation between pharyngeal residue volume and adventitious sounds. The results of this study are expected to be drawn by 2025 upon its completion. This study will demonstrate the feasibility of AI-based acoustic monitoring for quantifying increased pharyngeal residues during perioperative management. This approach has the potential to reduce the risk of postoperative aspiration with a simple and inexpensive method.

## Introduction

Swallowing dysfunction is frequently observed immediately after general anesthesia because of the residual effects of anesthetics, narcotics, and postoperative pain [1]. Consequently, pharyngeal residues often increase. Meta-analyses of the American Society of Anesthesiology Closed Claims database indicate a rising trend in perioperative complications, particularly aspiration following extubation [2]. Considering that this tendency persists despite advancements in anesthetic agents, muscle relaxants, airway management devices, and monitoring instruments [3], the current airway management protocols and monitoring methods may not be adequate to prevent postoperative aspiration. We hypothesized that detecting an increase in pharyngeal residue (e.g., saliva or blood) would alert healthcare professionals to an elevated risk of aspiration, enabling timely interventions such as suctioning. Therefore, we developed an electronic auscultation system capable of detecting such pharyngeal residues [4,5].　

In a previous investigation conducted using a prototype system, the adventitious sound index increased when 5 cc of fluid was instilled into the oral cavity of 60 patients undergoing monitored anesthesia care [6], and this trend was more pronounced in patients with obesity [7]. Nevertheless, as the actual pharyngeal residue was not verified during the intervention, the correlation between the pharyngeal residue and adventitious sound index has not been conclusively demonstrated [6]. Therefore, we designed a single-arm comparative trial protocol to clarify the correlation between the newly developed adventitious sound index and pharyngeal residues and validate the use of the adventitious sound index to observe increases in pharyngeal residues as an early indicator of aspiration.

## Materials and methods

Ethical considerations

This study was approved by the Hiroshima University Certified Review Board (approval number: E2024-0025) and registered at UMIN (UMIN000055020). Written informed consent will be obtained from all patients or their guardians.

Study design

The first draft of the study protocol was finalized on April 18, 2024, following the Standard Protocol Items: Recommendations for Interventional Trials (SPIRIT) Reporting Guidelines. Table 1 presents the primary and secondary objectives of this study. The primary endpoint of this study is to identify the association between pharyngeal residue levels and the adventitious sound index. In addition, we aim to clarify the feasibility of monitoring aspiration risk using the newly developed AI-based adventitious sound analysis system. The secondary endpoints are to verify the correlation between the pharyngeal residue volume and adventitious sounds. 

**Table 1:** Primary and secondary endpoint.

Endpoints	Details
Primary	Acoustic sensors will placed on the neck and chest to compare the adventitious sound index before and after aspiration of pharyngeal residues at each location.
Secondary	(1) Correlation between the mass of pharyngeal residue and the adventitious sound index. (2) Acoustic sensors will be placed on the neck and chest to compare the adventitious sound index before aspiration of pharyngeal residues at each location.

Sample size calculation 

A single-arm, pre-post comparison study was previously conducted among 19 patients with obesity who underwent anesthesia monitoring using an acoustic monitoring device. The adventitious sound index was measured before and after intraoral water injection during monitored anesthesia. The mean ± standard deviation of the indeterminate sound INDEX before and after water injection was 0.0679 ± 0.056 and 0.1415 ± 0.111, respectively. Based on these measurements, an effect size of 0.58 was calculated using G*Power software (version 3.1.9.6, Heinrich-Heine-Universität Düsseldorf, Düsseldorf, Germany). Using a two-sided test at a 5% significance level, 26 participants are required to maintain 80% statistical power. After accounting for an anticipated dropout rate of approximately 10%, a final sample of 30 participants is deemed necessary.

Inclusion and exclusion criteria

**Figure 1:**
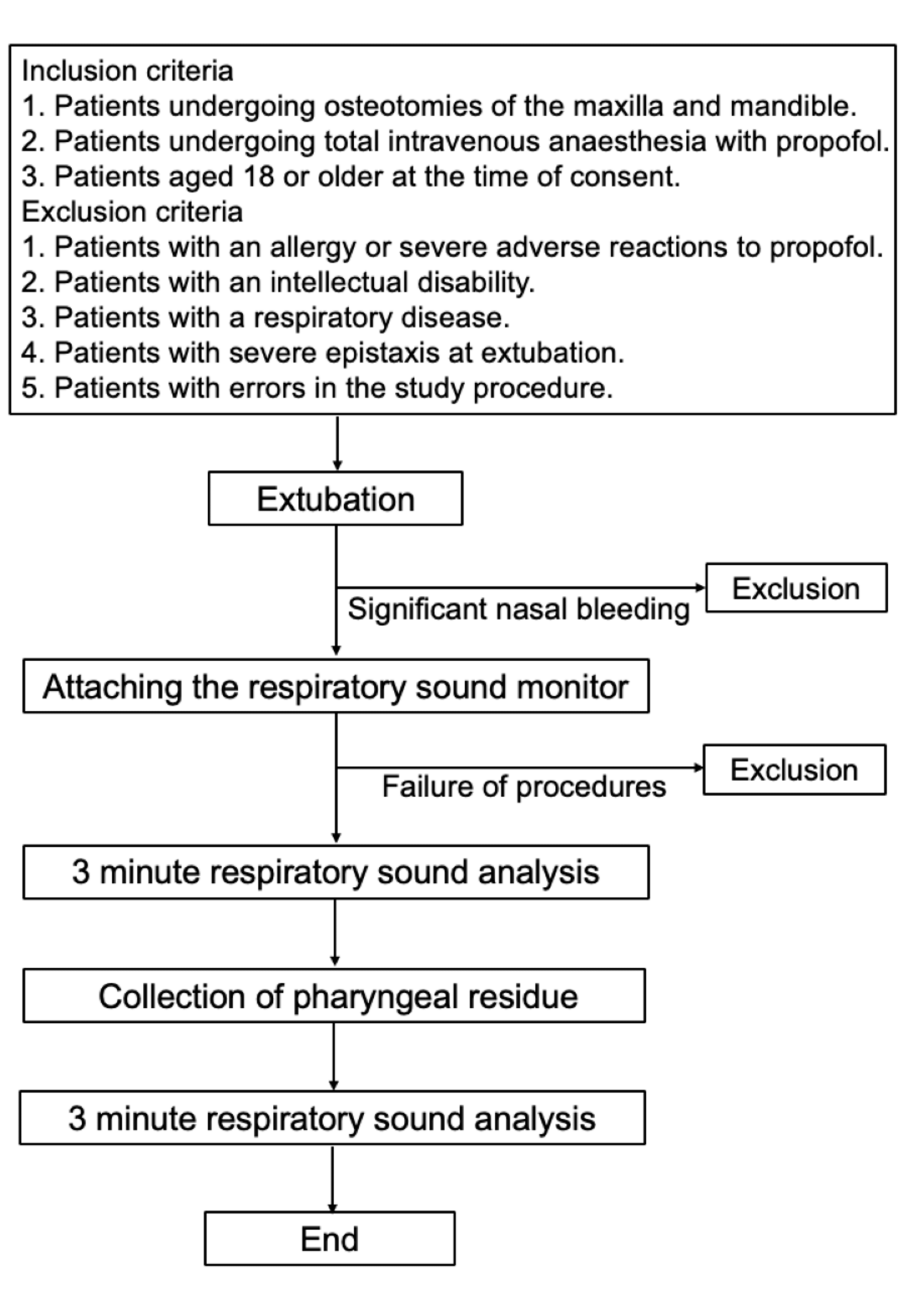
Schema of the study protocol.

We will recruit patients who are scheduled to undergo osteotomy of the upper and lower jaw for osteodeformity. The detailed inclusion and exclusion criteria are presented in Figure 1.

Table 2 shows the SPIRIT schedule for patient enrollment, interventions, and assessments.

**Table 2:** SPIRIT schedule of enrollment, interventions, and assessments. Table 2 shows the SPIRIT schedule for patient enrollment, interventions, and assessments.

Study period	Pre-Enrolment	Enrolment	Post-Enrolment	Close-out
Time point	-1 day	0 day	+1 day	
Enrolment	X			
Eligibility screen	X			
Informed consent	X			
Respiratory sound monitoring		X		X
Collection of pharyngeal residual fluid		X		X
Residual fluid in the pharynx		X		X
Respiratory sound analysis		X		X

Procedure

Propofol, remifentanil or fentanyl, and rocuronium will be used for general anesthesia. Intraoperative monitoring includes electrocardiography, oxygen saturation measurements, capnography, bladder temperature measurements, electroencephalography, and neuromuscular function measurements. Extubation during emergence will be performed once the train-of-four (TOF) ratio and the Entropy index (GE Healthcare’s Brain Monitoring System) have recovered to at least 90% and it is confirmed that the patient can open their eyes, open their mouth, and elevate their tongue.

**Figure 2:**
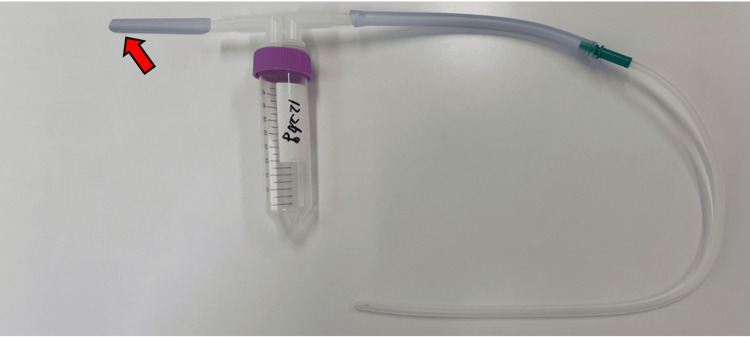
Containers for collecting residual pharyngeal fluid. Red arrow: Connect this end to the suction device.

The study procedure is illustrated in Figure 1. Immediately after extubation, the respiratory sound monitoring device will be attached to the skin of the neck (paralaryngeal region) and chest (beneath the sternal notch) using an adhesive tape (TransporeTM, White, 3M, USA). Subsequently, respiratory sound sampling will be initiated. After three minutes of sampling, the residual pharyngeal fluid will be suctioned using a McGrathTM MAC videolaryngoscope (Aircraft Medical Ltd., Edinburgh, UK), and an additional three minutes of respiratory sound recording will be performed. The residual pharyngeal fluid will be aspirated into a container (Triple Seal CapTM, AGC Techno Glass Co., Shizuoka, Japan; Figure 2), and after suctioning, the residual material inside the suction tube will also be flushed using 20 mL of normal saline. The weight of the pharyngeal residual substance will be measured as the change in the weight of the container before and after fluid collection.

Data collection

Two anesthetists will confirm the clinical evaluation and anesthetic records, and the following data will be recorded: height, weight, sex, degree of obesity, presence of respiratory disease, presence and degree of sleep apnea, Cormack-Lehane grading, presence of dysphagia, history of head and neck surgery, and presence of serious allergies. Six anesthesiologists will collect the auscultation data, and the presence or absence of profuse nasal bleeding and compliance with monitoring procedures will be recorded. 

The auscultation data will be stored as comma-separated value (CSV) files generated at one-second intervals from AI-based analyses, and these data will be cross-referenced with the corresponding WAV audio files. Within the three-minute recording periods before and after pharyngeal suctioning, auscultation segments with minimal noise and patient movement will be identified. Among these segments, we will determine the segments intended as reference intervals for the comparison of adventitious sound indices. If sensor detachment, application freezing, or noncompliance with sampling procedures for pharyngeal residue is observed during data acquisition, data collection is discontinued.

Statistical analysis

The 30-second mean values of the adventitious sound index measured before and after suction will be calculated, and a paired t-test will be performed for each of the three categories of the index. To determine the superior monitoring location, the index values obtained before suction will be subjected to analysis of variance for each type of index at both the cervical and thoracic monitoring sites. Statistical significance will be set to p < 0.05. significant. All statistical analyses will be conducted using SPSS v. 25 for Mac (IBM Corp., Armonk, NY).

## Results

This study will commence in January 2025 at the Hiroshima University Hospital, and the results will be obtained after completion of the study.

## Discussion

In our previous single-cohort intervention study, we demonstrated that the adventitious sound index responded significantly when water was instilled into the oral cavities of patients managed under monitored anesthesia care. However, this previous study did not confirm whether the instilled water actually accumulated in the pharynx [6]. Therefore, using the present protocol, we plan to clarify the correlation between the adventitious sound index and pharyngeal residue by collecting and weighing the pharyngeal residue immediately after awakening from general anesthesia using a video laryngoscope. We will also compare the adventitious sound index before and after this procedure. Using this approach, we expect to elucidate the correlation between pharyngeal residues and adventitious sound indices.

In our previous studies, the sensors for respiratory sound monitoring devices were positioned in the peripharyngeal region [6,7], and increases in the adventitious sound index observed at this site were presumed to result from airflow alterations synchronized with inspiration driven by the pharyngeal residue. However, adventitious sounds have also been identified as arising from bubbling sounds related to intratracheal secretions [8]. Consequently, we could not exclude that the increased adventitious sound index detected in the neck region may have been produced by intratracheal secretions rather than pharyngeal residues. In the current study, we plan to place sensors on both the neck and chest. By comparing the adventitious sound indices at these two anatomical sites, we anticipate that the origin of adventitious sounds could be precisely identified.

Our study group reported that the respiratory sound monitoring system used in this study can quantify the level of adventitious sounds generated in the pharyngeal region by the infusion of water into the oral cavity [9-11]. Furthermore, it has been reported that the adventitious sounds index utilized by this AI system demonstrates approximately 80% sensitivity and specificity relative to physicians’ auscultatory assessments [4]. The adventitious sounds index is calculated at a rate of one frame per 12 milliseconds, enabling real-time and multi-channel monitoring of these sounds. It is anticipated that this system could be widely utilized for aspiration risk monitoring in various clinical situations. 

The acoustic sensor employed in the process is a cost-effective displacement-detection vibration sensor. By clarifying the sensor installation requirements necessary for identifying pharyngeal residues through acoustic monitoring, a novel evaluation method for quantifying the perioperative aspiration risk may be established, it is envisaged that the device will establish a widely applicable pharyngeal residue assessment index. Nevertheless, several limitations are identified in the method used to validate this research. First, the collected liquid residue contains various substances, including blood, saliva, and mucus. Although changes in weight serve only as a quantitative indicator, it remains unclear which types of fluid pose the aspiration risk. Furthermore, the findings may not be generalizable to surgeries other than those involving the head and neck. Second, the evaluation of pharyngeal residue using the adventitious sounds index is subject to confounding factors, including obesity, upper airway obstruction such as sleep apnea, and underlying conditions like tonsillar hypertrophy. In particular, a correlation has been reported between obesity, body mass index (BMI), and the adventitious sounds index [7]. These confounding factors will be documented, and a multivariate analysis will be performed. Third, the inability to restrict the patient’s swallowing has been identified as a concern. When the pharyngeal residue is swallowed by the patient, the amount of residue is inevitably reduced. Fourth, it should be noted that the adventitious sounds index can be increased by environmental noise, as well as by the patient’s own vocalization or coughing.

In another study, pharyngeal residue was evaluated by attaching an acoustic sensor to the neck (paralaryngeal region) to measure adventitious sounds [12,13]. Although paralaryngeal auscultation with a traditional analog stethoscope is commonly employed in clinical practice to detect pharyngeal residue, it is necessary to exclude or consider the possible influence of adventitious sounds from the lungs when performing automatic respiratory sound analysis [14,15]. Therefore, in the present study, auscultation of the chest (at the upper sternal border) will also be conducted, and the magnitudes of the adventitious sound indices obtained from both sites will be compared. Lung diseases are added to the exclusion criteria, and the influence of adventitious sounds transmitted from the lungs is eliminated by performing endotracheal suctioning before extubation. By doing so, we can specifically infer the adventitious sounds arising solely from pharyngeal residue.

## Conclusions

This study will demonstrate the feasibility of acoustic monitoring as a newly developed evaluation index for quantifying increased pharyngeal residues caused by impaired swallowing function during perioperative management. Using AI-based acoustic monitoring to simply and inexpensively evaluate the risk of aspiration, this approach may be able to reduce the risk of postoperative aspiration.
